# Optically isotropic fast phase modulation in 3D blue phase photonic crystals

**DOI:** 10.1038/s41598-025-25918-0

**Published:** 2025-11-25

**Authors:** Tomasz Jankowski, Eva Oton, Noureddine Bennis, Anna Pakuła, Przemysław Morawiak, Wiktor Piecek

**Affiliations:** 1 Faculty of New Technologies and Chemistry, Military University of Technology, Warsaw, Poland; 2 Faculty of Mechatronics, Warsaw University of Technology, Warsaw, Poland

**Keywords:** Blue phase liquid crystals, Photonic crystals, Phase modulator, Polarization independence, Materials science, Optics and photonics, Physics

## Abstract

Blue Phase (BP) liquid crystals are self-assembled 3D photonic structures that uniquely combine optical isotropy with ultrafast electro-optical switching. Their cubic symmetry enables polarization-independent behavior, while sub-millisecond response times make them highly attractive for dynamic photonic applications. However, their practical use has typically been limited by their tendency to form disordered polycrystalline domains, resulting in scattering and poor optical performance. In this work, we realized large-area monocrystalline BP structures with a photonic stopband positioned entirely outside the visible range. This eliminates structural coloration and enables a fully transparent, broadband operation without parasitic reflection. The cubic symmetry ensures the system remains optically isotropic, supporting polarization-independent modulation. We demonstrate a fast phase modulator based on this monocrystalline BP platform. The device achieves phase modulation with sub-millisecond response times and polarization independence, representing a significant advancement over traditional LCs. Our findings position monocrystalline BPs as a robust and versatile platform for reconfigurable optics. The combination of fast response, polarization independence, and optical transparency opens new pathways for advanced applications in adaptive optics, beam steering, dynamic holography, and transparent optical systems such as AR/VR and laser-based technologies.

**Supplementary Information:**

The online version contains supplementary material available at 10.1038/s41598-025-25918-0.

## Introduction

Blue Phase (BP) liquid crystals represent a remarkable class of self-assembled photonic materials that combine the structural order of 3D photonic crystals with the electro-optical responsiveness of soft matter^1^. Their cubic structure—body-centered cubic (BPI) or simple cubic (BPII)—induces optical isotropy due to symmetry of the unit cell, setting them apart from conventional anisotropic liquid crystal systems such as nematics or cholesterics and adding attractive photonic properties^2^. Moreover, BPs are uniquely suited for fast modulation applications owing to their sub-millisecond switching speeds under applied electric fields^3^.

A critical limitation to the practical use of BPs has long been their tendency to form disorganized polycrystalline structures: conventional BP crystals form randomly oriented platelet domains that significantly degrade device performance. These polycrystals consist of small, misaligned BP domains that scatter light, introduce optical anisotropies, and produce poor electro-optical behavior^4,5^. Addressing this issue requires the development of new methods for producing large, single-domain BP monocrystals with controlled lattice orientations, and various solutions have been reported, like alignment layers, photopatterning, or structured surfaces^6–9^.

Advances in our group with materials design and surface anchoring techniques, however, have enabled the fabrication of large-scale (mm-sized) BP monocrystals with controlled lattice orientation. The resulting monocrystalline BP structures exhibit superior optical properties. The alignment and azimuthal orientation of the cubic lattice are governed by surface anchoring conditions, where interfacial free energy selectively stabilizes specific lattice configurations^10^. In this work, we build upon these strategies to fabricate large, optically uniform BP photonic crystals, dramatically improving the optical quality and functional uniformity of BP-based devices.

In this work, we manufactured large stabilized BP photonic crystals with a photonic stopband located entirely outside the visible range. This feature removes the Bragg reflection in the visible spectrum—an issue that typically limits the practical utility of BPs in transparent optical devices. More importantly, the cubic symmetry of the crystal ensures that the structure remains optically isotropic, enabling operation that is completely independent of input polarization.

An additional key characteristic of BPs is their rapid electro-optical response, with electric field-induced switching occurring on microsecond timescales. This ultrafast modulation behavior makes BPs highly suitable for applications demanding swift and dynamic control of optical signals^11,12^.

We use this combination of properties to demonstrate a fast polarization-independent phase modulator, operating in a spectral range outside the visible range, thus eliminating unwanted structural coloration and enabling integration into broader optical systems without parasitic reflection. The phase modulator exhibits sub-millisecond response times, enabled by the intrinsic fast switching behavior of BPs.

This represents a significant improvement for BP-based photonics. Unlike conventional LC modulators that require precise alignment and suffer from polarization sensitivity, our system provides a robust and broadband platform for high-speed, polarization-independent phase control. The combination of isotropy, speed, and transparency unlocks new applications, including adaptive optics, beam steering, dynamic holography, and transparent optical components for AR/VR and laser systems.

Our findings demonstrate that when structural disorder is eliminated and the photonic bandgap is tailored appropriately, BPs provide an unparalleled platform for fast and isotropic phase modulation, which opens up new pathways in the development of reconfigurable systems. This marks a significant step toward high-performance, fast-response, and robust photonic components for adaptive optics, beam steering, and beyond.

## Results

### Stabilizing large BP monocrystals

**Fig. 1:**
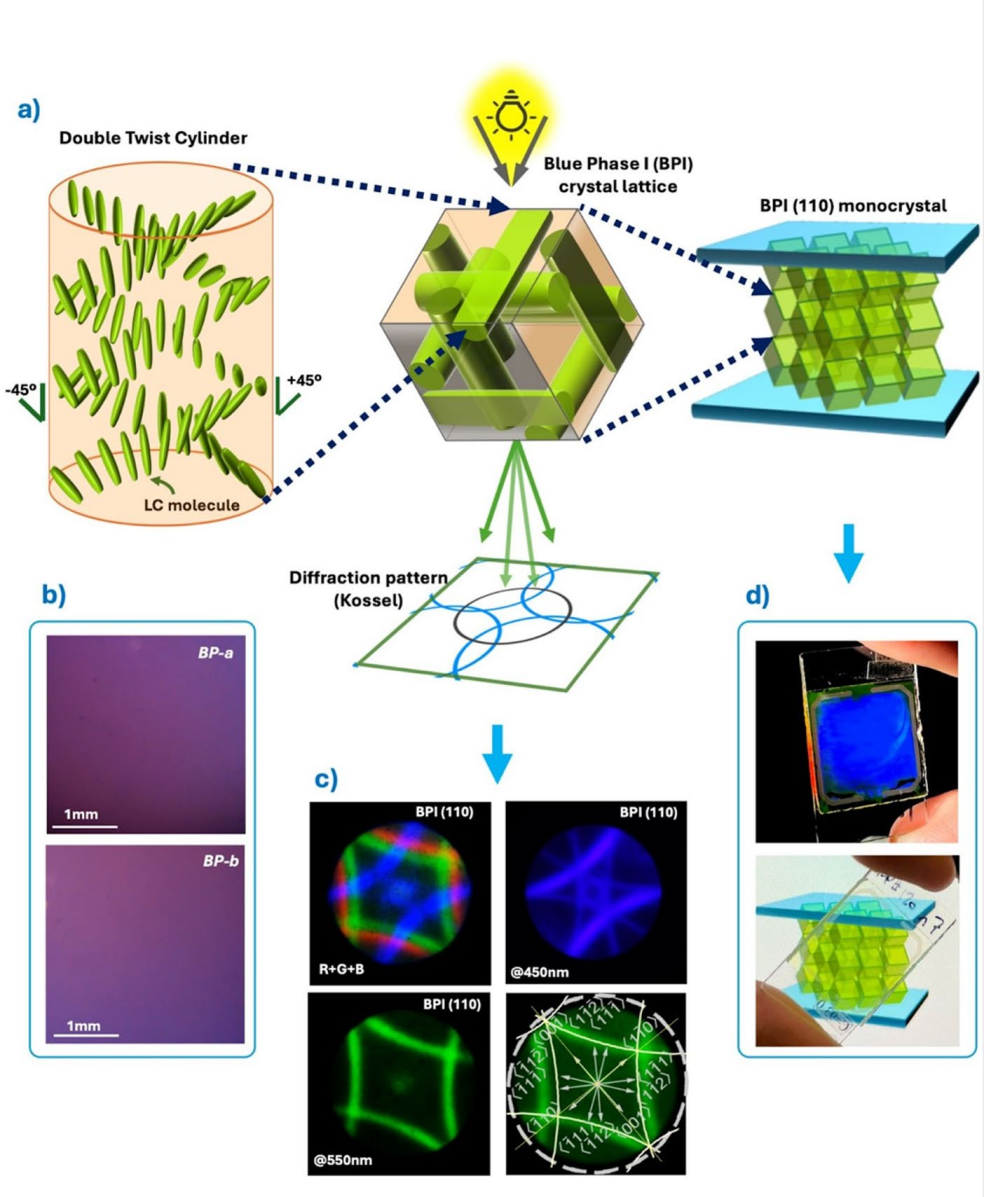
Formation of a blue phase monocrystal: (**a**) (from left to right) double twist cylinder self-assembling into a blue phase I (BPI) lattice structure, BPI crystal lattice and diffraction pattern (Kossel), and a fully stabilized BPI monocrystal in lattice orientation (110). (**b**) Stabilized BPI (110) textures for both BP mixtures (scale 1 mm). (**c**) Corresponding Kossel patterns observed with a 3-channel RGB LED source, 450 and 550 nm, and Kossel pattern line identification for BPI (110). (**d**) Side Bragg reflection and transmitted light of a stabilized BP monocrystal in a large cell observed by white LED illumination.

The formation pathway of a BP monocrystal is outlined in Fig. 1a. Upon cooling from the isotropic phase, LC molecules spontaneously assemble into double-twist cylinders (DTCs), where the director twists simultaneously in two directions. This configuration minimizes local elastic free energy but cannot uniformly fill space, leading to the emergence of a three-dimensional periodic network of disclination lines.

In BPI, the DTCs organize into a body-centered cubic (BCC) lattice. This cubic periodicity produces selective Bragg reflection of visible light, giving rise to striking iridescent colors. When the lattice nucleates and grows with a single crystallographic orientation across the surface-treated sample volume, a BP monocrystal is obtained. Such monocrystals exhibit macroscopic long-range order, a prerequisite for advanced optical applications.

To extend structural stability beyond the intrinsically narrow BP temperature range, the monocrystals were polymer-stabilized. This was accomplished by incorporating reactive monomers into the LC mixture, which preferentially polymerize along disclination lines and mechanically lock the DTC lattice into place (see Methods).

The lattice parameters were selected by adjusting the precursor mixture concentrations to obtain the desired BP lattice orientation and lattice size in each case, following the methods developed in our previous study^10^.

The uniformity and lattice orientation of BP monocrystals were analyzed by polarized optical microscopy (POM) and Kossel pattern analysis. A convergent light beam passing through a BP monocrystal will show a diffraction pattern related to the crystal periodicity (Fig. 1a). These patterns, known as Kossel patterns, can be thought of as similar to X-Ray diffraction patterns but with a scale factor – given that the BP lattice periodicity is in the order of hundreds of nm^13^.

Two LC base mixtures were prepared in a range that we know induces monocrystalline BPI^10^. The mixtures were designed to induce a BPI with lattice orientation (110) whose reflection band will fall outside the visible range (λ > 700 nm). Both mixtures, *BP-a* and *BP-b*, were prepared to contain a host liquid crystal mixture base, a chiral dopant, and a mix of monomers. The main host components for both BP precursor mixtures are identical (see Methods), but they each contain a different chiral dopant with opposite-handedness at 5.3% (*BP-a*) and 5.0% (*BP-b*). The precursor mixtures were then filled into surface-treated sandwich-like cells in isotropic state and then stabilized into large, mm-sized BP crystals.

The obtained BPs were analyzed by POM (in reflection) and Kossel analysis: Fig. 1b shows the textures of both BP monocrystals obtained from mixtures *BP-a* and *BP-b*. The reflection color was not accurately caught by our CCD camera (very dark, being in the IR range); however, the texture is clearly homogeneous with the same reflection color and no defects. The monocrystal nature of BPs can be revealed by their Kossel patterns. Figure 1c shows the Kossel patterns for *BP-a*, observed with a 3-channel RGB LED source, a 450 nm, and a 550 nm source. The diamond-shaped pattern indicates the BP crystal BPI and is oriented in the BPI (110) crystal lattice orientation. The patterns remain sharp along the whole sample with no rotation, blurry lines, or spatial variations. This demonstrates that the BP crystals are indeed monocrystals with a fixed lattice orientation because mixed or blurry lines indicate a loss of crystallinity and a decreased order degree in the BP crystal structure^13^. The Kossel pattern line identification for BPI (110) is shown in the fourth Kossel diagram. Kossel lines for *BP-b* were equivalent to *BP-a*.

BP crystals’ 3D periodicity shows selective reflection in three dimensions, so a white light shone on the BP cells’ edge produces an amplified bright side Bragg reflection shining through the surface of the cell glasses. Figure 1d (top) shows a stabilized BP crystal in a cell being illuminated from the edge. A bright blue reflection is seen at high angles with respect to the glass surface. Because the reflected light is amplified back to the surface, this method allows for a quick assessment of the crystal’s quality. Misoriented areas or defects will appear as dark areas, scattering, or haze. In this case, the BP crystal shows a uniform reflection color, demonstrating a single monocrystal. Figure 1d (bottom) shows the same sample while being illuminated from the back; the BP crystal cell has high transparency in the whole visible range.

All experiments were conducted at room temperature. The polymer-stabilized BP monocrystals displayed remarkable structural resilience, consistently preserving their lattice orientation and optical response under repeated high-power illumination. Additional stability tests confirmed that the monocrystals remained intact over a broad temperature range (0–55 °C), far wider than the narrow window of native, non-stabilized BPs. Even after multiple thermal cycles and prolonged high-power irradiation, no degradation of Bragg reflection intensity or peak wavelength was detected. These results highlight the robustness of the monocrystals and their reliability for stable photonic operation under realistic conditions.

### Polarization independency

**Fig. 2:**
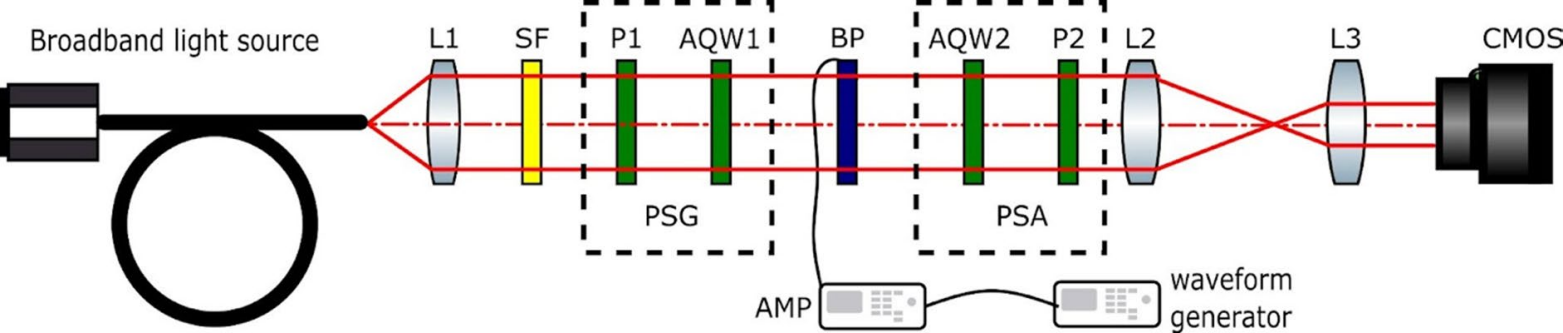
Optical system for measuring BP polarimetric response.

To study the dynamics of the BPs, we addressed them with a waveform generator producing a 1 kHz square wave voltage pulse in conjunction with a high-voltage amplifier (AMP) to generate voltages up to 200 V. The electro-optical properties were obtained using a specific optical system setup shown schematically in Fig. 2. We employed an unpolarized broadband light source that was spectrally filtered in the range [400–700 nm] and collimated by lens L1. A polarization state generator (PSG) consisting of a linear polarizer (P1) and an achromatic quarter-wave retarder (AQW1) was used to create horizontal (H), vertical (V), diagonal at 45° (+ 45°), right circular (CR), and left circular (CL) input states of polarization illuminating the BPs. Behind the samples, a polarization state analyzer (PSA) comprising another achromatic quarter-wave plate (AQW2) and a linear polarizer (P2) was used to analyze the output beams. To detect small polarization-induced intensity changes, the system worked in parallel PSG and PSA configurations. Additionally, a two-lens telescope system (L2 and L3) is implemented to image the output beam onto a CMOS camera. Before conducting measurements, reference images were captured without the sample in the system to assess the beam’s intensity for all SOPs.

**Fig. 3:**
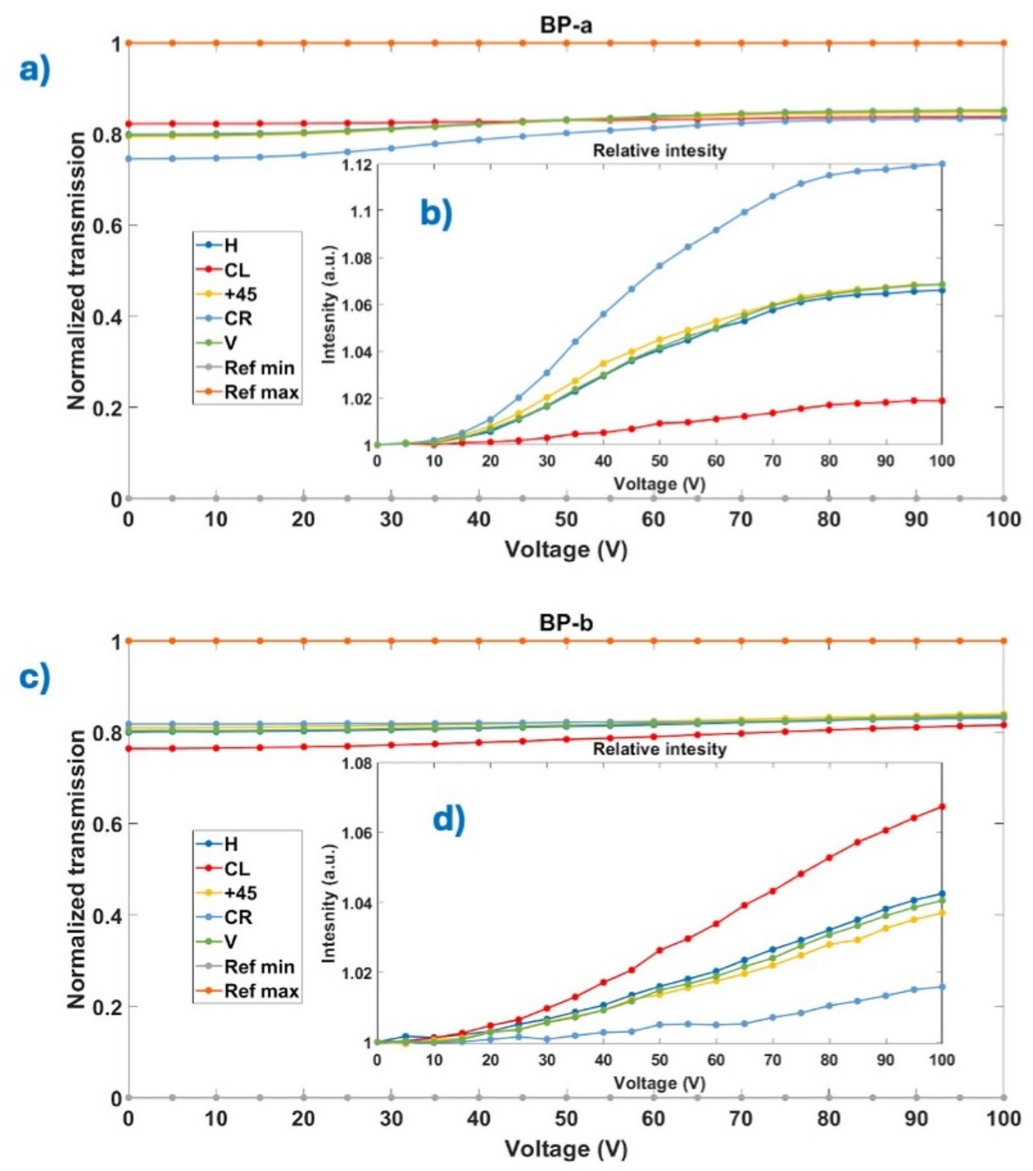
Normalized transmission of both kinds of BPs with insets of relative intensity for: (**a**) BP-a, and (**b**) BP-b. A two-step normalization was performed: the first step (**a**,** b**) calculates the ratio of measured values to the reference values, and the second (**c**,** d**) further relates these results to the values in the BP in the off state. Achieving a minimum intensity of 1 in each data series helps in analyzing relative intensity variations.

The polarization independence of the BP structure was evaluated through voltage-dependent scattering loss measurements. As shown in Fig. 3, measurable scattering losses are observed, particularly just below the electro-optic threshold voltage. This behavior is consistent with prior reports on stabilized BPs, where minor scattering arises due to refractive index mismatches between the liquid crystal host and the supporting polymer network that maintains the photonic crystal structure^5^. Upon application of an increasing electric field, reorientation of the BP structure reduces these small index mismatches, thereby suppressing scattering and improving optical transmission. Both kinds of BP crystals (*BP-a* and *BP-b* with opposite chirality) show almost identical results.

Essentially, scattering measurements were performed across a full set of linear input polarizations. The results show that the small scattering losses are effectively invariant with respect to input polarization state, indicating that light interacts with the BP lattice in a manner independent of polarization. Naturally, this confirms the intrinsic optical isotropy of the BP structure, as dictated by its cubic symmetry. The uniformity of scattering across polarization states provides strong experimental evidence for the polarization-independent behavior of monocrystalline BPs, reinforcing their suitability for applications requiring robust and alignment-free modulation of unpolarized or arbitrarily polarized light.

### Electrooptical measurements

Response time measurements were conducted using the polarimetric setup described previously, with the CMOS imaging system replaced by a high-frame-rate photodetector to enable accurate capture of the fast electro-optic dynamics characteristic of BPs. To accommodate the sub-millisecond switching behavior, the driving voltage waveform was adapted to a bipolar square pulse sequence: two pulses of equal duration (2.5 ms) and opposite polarity, separated by a 2.5 ms *off*-interval. The voltage amplitude was incrementally increased during the measurements, reaching a maximum of 100 V—well above the voltage threshold.

**Fig. 4:**
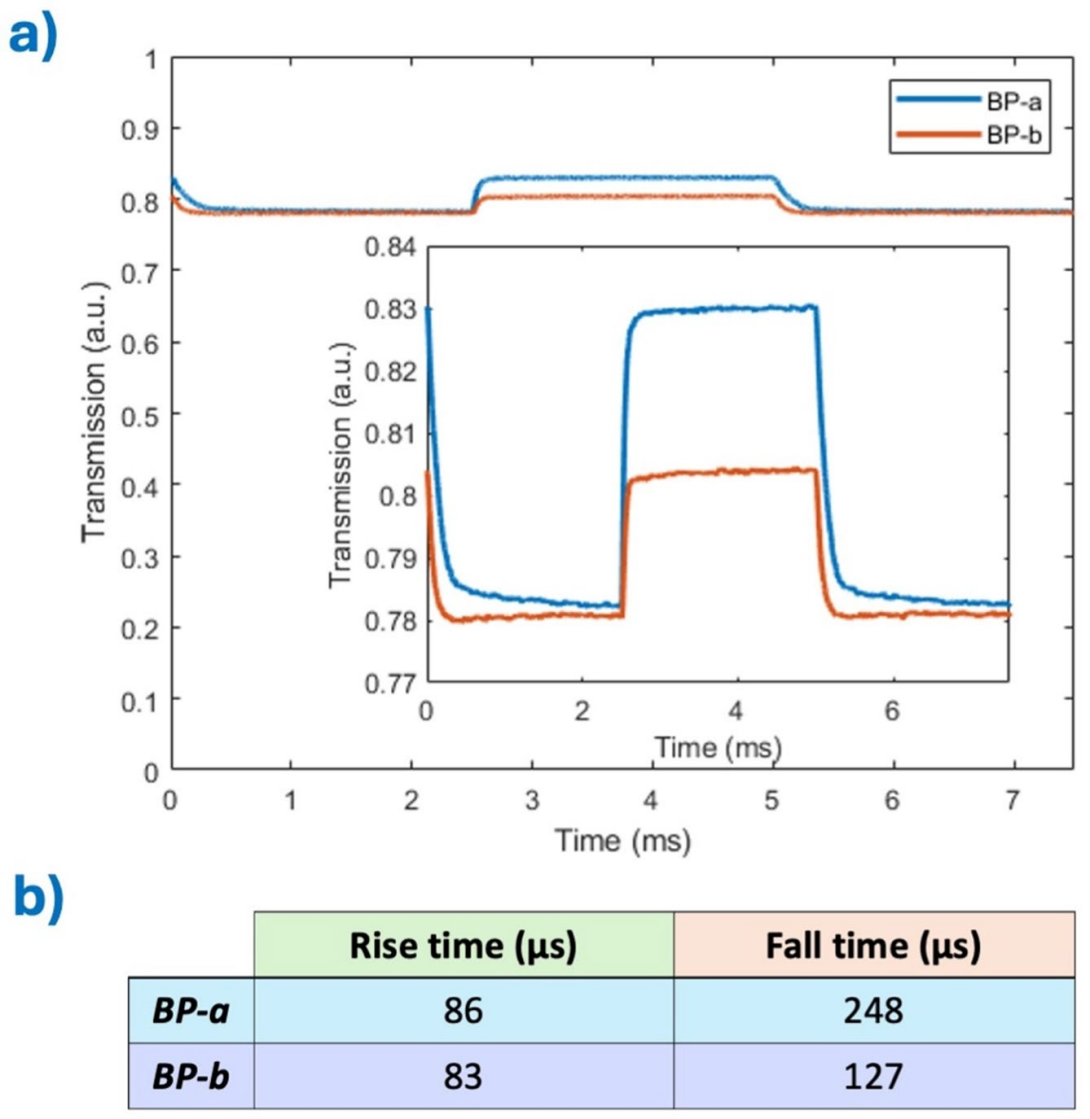
Electro-optical measurements, (**a**) transmission curve vs. time for BP-a and BP-b, (RT = rise time, FT = fall time), (**b**) calculated response times.

Figure 4a presents the dynamic optical response of the BP monocrystals, and the extracted response time values are summarized in Fig. 4b. Both compositions exhibit exceptionally fast rise times, consistent with the intrinsic rapid switching capabilities of BP crystals. Notably, the rise time remains sub-millisecond across all conditions tested. While both materials demonstrate comparable rise dynamics, *BP-a* exhibits a longer fall time, approximately twice that of *BP-b*, suggesting material-specific relaxation pathways post-field removal. We attribute this behavior to the different chiral dopants with opposite-handedness used in the precursor mixtures.

Furthermore, it is important to note that the observed transmission changes in the BP crystals are relatively modest. This is primarily because the operating wavelength used in our measurements lies well outside the photonic stopband of the BPs, where Bragg reflection effects are minimal. As a result, the modulation of transmitted intensity arises predominantly from subtle changes in refractive index anisotropy rather than strong photonic bandgap interactions. Additionally, BP crystals possess a relatively low birefringence and an overall effective refractive index that is close to that of the surrounding medium, typically around 1.6. This low refractive index contrast further limits the amplitude of transmission modulation.

Despite these constraints, the phase modulation remains substantial due to the coherent electro-optic response of the periodic structure, emphasizing the material’s suitability for applications that rely on fast phase shifts rather than amplitude changes. These results underscore the ultrafast response of monocrystalline BPs, a key advantage for high-speed optical modulation applications where rapid and repeatable switching is essential.

### Phase modulation of the BP crystals

**Fig. 5:**
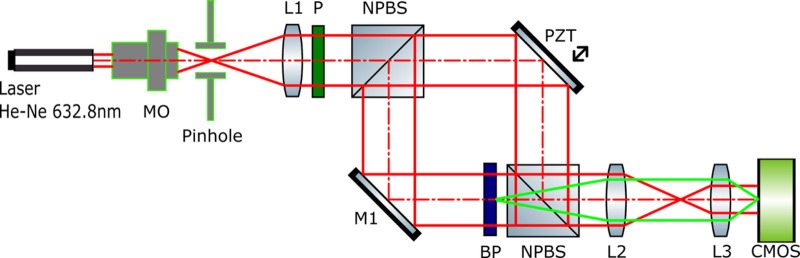
Mach-Zehnder interferometer set-up used for measuring the BPs phase modulation.

The phase modulation responses of the BPs were tested using a full-field Mach-Zehnder interferometer (Fig. 5) using a collimated beam (MO, Pinhole, L1) from a He-Ne laser, which is polarized by a polarizer (P). The beam is then divided into two branches by a non-polarizing beam splitter (NBPS). In the object branch, a mirror (M1) is positioned, while in the reference branch, a second mirror is mounted on a piezo-transmitter (PZT). The mirrors redirect both beams to a second NBPS, forming an interference pattern. This pattern is subsequently resized using two lenses (L2, L3) and detected by a CMOS camera. The tested sample is positioned so that its optical plane is conjugated with the camera plane. For phase measurement, the Temporal Phase Shift (TPS) method was employed for wrapped phase extraction. The method’s error is estimated to be λ/20 = 0.31 rad for the phase value at a single point in the active area at the specified voltage. The uncertainty of the mean phase was calculated using the standard uncertainty for the mean. The total phase extraction error was determined to be ± 0.02 rad, based on the standard uncertainty of the mean and type B measurement uncertainty (see Methods section).

**Fig. 6:**
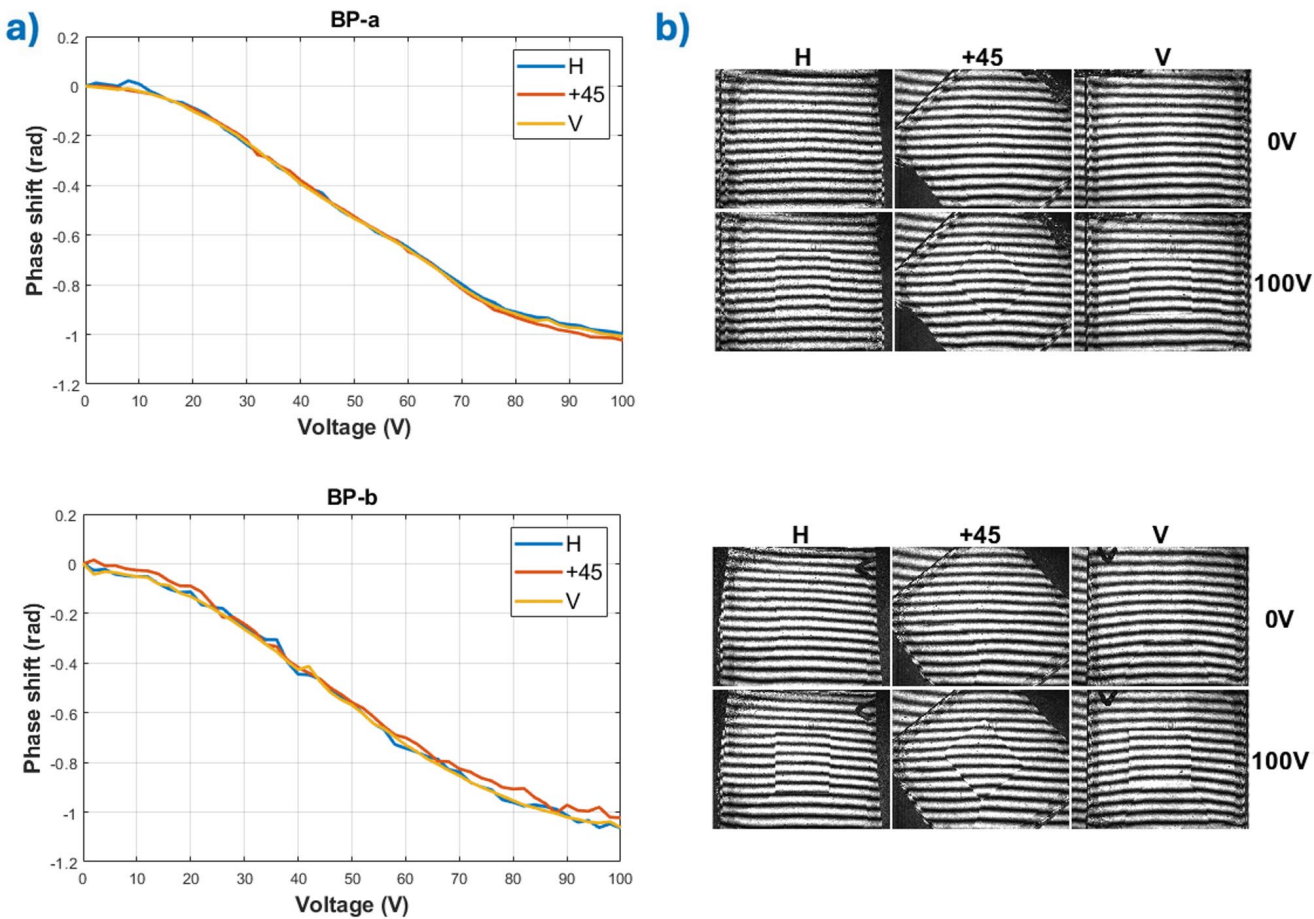
Interferometric measurements for both materials: (**a**) phase modulation for BP-a and BP-b with different polarization states of the incident beam, (**b**) interferograms for BP-a and BP-b, with and without applied voltage for horizontal, vertical, and + 45º.

To quantitatively assess the phase modulation performance and polarization independence of the BP crystals, three measurement sets were acquired for each sample. Between each set, the sample was rotated by 45°, resulting in three distinct orientations of the incident linear polarization state. Phase retardation was determined relative to the zero-field state for each dataset. Figure 6a shows the phase modulation for *BP-a* and *BP-b*. From the measure response curve, phase saturation was achieved before our maxi6mum voltage input. Both threshold and saturation voltages for both types of BPs were not significantly different from each other at a fixed cell thickness. The actual interferograms for three different polarization states are shown in Fig. b, where the photographs when the voltage is applied clearly show the active area of the devices by the shifted interference lines.

Each phase shift measurement was conducted using all polarization states of the incident laser beam. In all cases, the electro-optical response and the maximum achieved phase shift were nearly identical for both polarizations, with negligible differences. The voltage-dependent phase modulation exhibits remarkable consistency across all tested input polarization angles, confirming the optical isotropy and polarization-independent behavior intrinsic to the cubic symmetry of monocrystalline BPs. This invariance with respect to input state of polarization is a critical advantage over conventional birefringent liquid crystal devices, which require precise alignment and are sensitive to polarization changes.

Moreover, both *BP-a* and *BP-b* samples exhibited a total voltage-induced phase shift of approximately 1.0 rad for 3 μm thick devices, and 1.6 radians for 5 μm devices (and a predicted π rad phase modulation for 10 μm devices), a significant modulation depth for such a compact device architecture. This level of phase retardation, combined with the fast switching dynamics and polarization insensitivity, highlights the suitability of monocrystalline BPs for broadband, alignment-free phase modulators in advanced photonic systems including adaptive optics, beam shaping, and holographic projection.

### Spectral measurements and Kossel analysis

Spectral measurements were complemented by a combination of POM, texture analysis, Kossel diagram evaluation, and reflection spectroscopy to verify the BP crystals’ stopband and lattice constants.

**Fig. 7:**
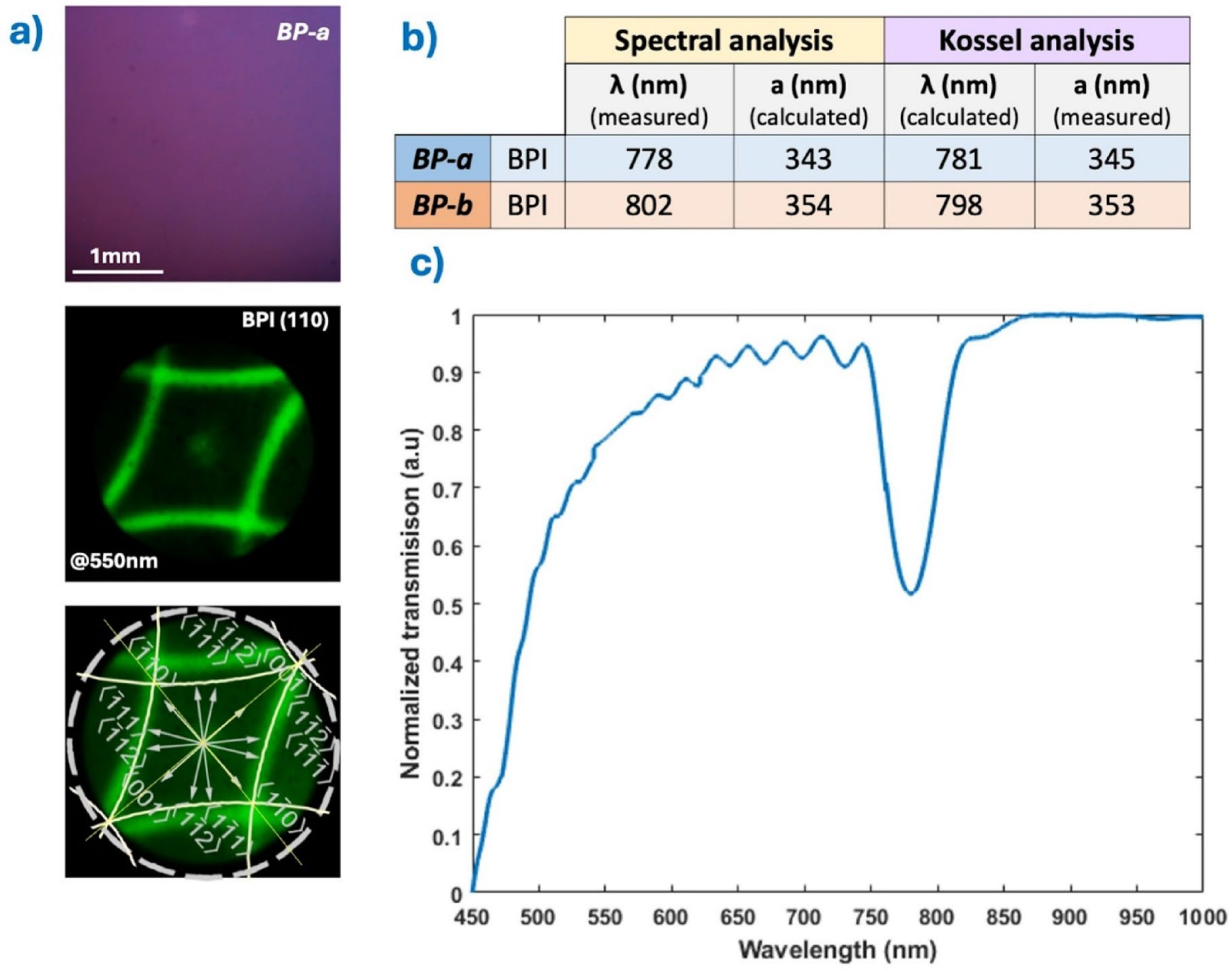
Spectral and Kossel analysis. (**a**) POM texture, corresponding Kossel pattern, and orthographic projections of the Kossel lines for BP-a. (**b**) Measured and calculated values of the lattice constant and reflection wavelength by Kossel analysis or spectral analysis. (**c**) Transmission spectrum for BP-a.

Lattice constants were estimated through both Kossel pattern analysis and reflection band measurements (Fig. 7a). From the experimentally obtained reflection spectra, the lattice size was determined based on the Bragg reflection condition for photonic crystals: λ = 2na/(√(h^2^ + k^2^ + l^2^)), where λ is the central wavelength of the reflection band corresponding to the Miller indices (*hkl*), *a* is the lattice constant, and *n* ≈ 1.6 is the assumed average refractive index of the BP medium.

Naturally, the initial BP bandgap can be readily tailored by adjusting the precursor mixture, by varying the concentration of chiral dopant, allowing the design of crystals with a desired photonic bandgap starting point while accounting for the helical twisting power (HTP). This makes the BP–chiral PD platform highly tunable, adaptable, and versatile^14^.

Kossel analysis was performed by simulating diffraction patterns of body-centered cubic (BPI) structures. The experimental Kossel lines (Fig. 7a) were matched to simulated orthographic projections generated for converging monochromatic light at 550 nm, taking into account the microscope objective’s numerical aperture. These simulations yielded precise lattice constant estimates, which were in good agreement with those derived from spectral measurements, as shown in the table in Fig. 7b.

Figure 7c shows the spectrographic measurements —confirming the reflection band is outside he visible range— for a *BP-a* crystal, where the maximum of the reflection band is λ_BP−a_ = 778 nm. The measurements for *BP-b* crystals are analogous with a reflection band centered at λ_BP−b_ = 802 nm.

## Discussion

The results presented here demonstrate the successful fabrication and optical characterization of a large-area 3D Blue Phase photonic crystal, exhibiting a photonic stopband centered well beyond the visible spectrum. This shift in reflection band—confirmed through both Kossel analysis and reflection spectroscopy—indicates a large lattice periodicity. The ability to achieve and stabilize BP monocrystals in selected lattice orientations over millimeter-scale areas is a significant advance over polycrystalline or disordered BP implementations. The Kossel patterns consistently showed sharp and invariant line structures across the entire sample area, attesting to the long-range order and single-domain nature of the BPI crystals.

An essential implication of the cubic symmetry in BPs is their intrinsic optical isotropy. Unlike nematic or smectic liquid crystals, which are inherently birefringent and highly polarization-dependent, monocrystalline BPs interact identically with all linear polarization states. This was experimentally confirmed through polarization-resolved measurements of both scattering loss and voltage-induced phase modulation. Across all tested polarization states, the scattering losses and phase retardation remained invariant, strongly supporting the polarization-independent behavior of the material. This property removes the need for polarization management or device aligning mechanisms, simplifying the design of optical devices.

Further, the electro-optical characterization reveals exceptionally fast switching dynamics, with rise times below 90 µs for both BP-a and BP-b formulations. This sub-millisecond response—over an order of magnitude faster than conventional nematic LC devices—positions BPs as ideal candidates for real-time modulation applications. Importantly, we measured phase delays of ~ 1.0 rad for 3 μm and ~ 1.6 rad for 5 μm-thick monocrystals at modest driving voltages (< 100 V). From these results, and considering the linear scaling of phase retardation with optical path length (Δφ = 2πΔn·d/λ), we can reliably predict that 10 μm-thick monocrystalline BP cells will yield phase shifts on the order of π radians.

Although our current setup limits us to thinner cells, these predictions are firmly supported by the excellent structural quality of our monocrystalline BPs. Unlike polycrystalline systems, which often exhibit haze and scattering that worsen with thickness, the uniform long-range order of our monodomains ensures that increased cell thickness does not compromise optical clarity or introduce alignment-related artifacts. Weak anchoring conditions, combined with precise control of chiral dopant concentration, consistently produced large-area, defect-free monocrystals, suggesting that thicker cells should retain the same level of optical performance.

Achieving a π-rad phase delay is a highly relevant milestone, as it enables full-wave modulation required for applications such as beam steering, adaptive optics, and spatial light modulators. Coupled with the intrinsic sub-100 µs response and polarization insensitivity of the cubic BP lattice, this strongly supports the case for monocrystalline BPs as a practical platform for next-generation high-speed, low-power, and alignment-free photonic phase modulators.

## Conclusions

In this study, we report the realization of stable, large-area monocrystalline BP photonic crystals with a well-defined (110) lattice orientation and photonic stopbands lying beyond the visible range. The confirmation of single-domain lattice structure through Kossel pattern analysis and reflection spectroscopy highlights the high degree of order in the fabricated samples. The resulting photonic crystals exhibit isotropic optical behavior as a direct consequence of the cubic symmetry of the BP lattice, enabling polarization-independent fast modulation.

Electro-optical measurements further confirm the excellent performance of these monocrystalline BPs as fast and efficient phase modulators. Phase shifts up to π radians were achieved uniformly across polarization states, and switching times were recorded in the sub-millisecond range, outperforming conventional LC technologies. These findings establish monocrystalline BPs as a compelling platform for next-generation photonic devices, particularly in applications requiring high-speed, broadband, and polarization-independent optical modulation, such as adaptive optics, beam steering, and holography.

## Methods

### BP mixture Preparation and monocrystalline BP crystal Preparation

BP precursor mixtures were prepared using an in-house formulation consisting of fluorinated terphenyls, biphenyls, and cyclohexylbiphenyls as the nematic host, combined with a blend of mesogenic monomers and the chiral dopant ISO(6OBA)₂ (Midori Kagaku Co. Ltd.). The concentrations were systematically adjusted to obtain the desired BP lattice orientation and lattice size, following the protocol established in our previous work^10^. There, we demonstrated that tuning the chiral dopant (CD) concentration together with weak anchoring surface conditions enables the fabrication of large BP monocrystals with precisely controlled orientation and reflection wavelength. Importantly, lattice orientation was found to depend sensitively on CD concentration even under identical surface conditions, suggesting that subtle variations in LC–substrate anchoring energy govern orientation selection. Weak anchoring was essential to achieve uniform monodomains, while conventional strong anchoring failed to produce comparable alignment. Phase transition studies revealed consistent, symmetry-governed orientation relationships between BPII and BPI, with certain transitions systematically absent, indicating crystallographic constraints between the simple cubic (BPII) and body-centered cubic (BPI) phases. By mapping lattice orientation against lattice size and reflection wavelength, we established a predictive link between CD concentration, helical twisting power, and BP orientation. This strategy was employed here to reproducibly obtain highly ordered BPI (110) monocrystals.

The mesogenic host mixtures used in this study contained ISO(6OBA)_2_ at 5.3% (*BP-a*) and 5.0% (*BP-b*) (see Fig. S1). All formulations were specifically designed to suppress BPI (200), which can coexist with BPI (110) and hinder the formation of single-domain BPI (110) monocrystals.

Glass cells were fabricated using indium–tin–oxide (ITO)-coated ultraflat substrates assembled into 3 and 5 μm thick sandwiches. The ITO layers were covered with alignment coatings, thermally conditioned, and rubbed in an antiparallel configuration. BP precursor mixtures were introduced into the cells by capillary action in the isotropic phase. After phase-transition analysis, the resulting BP monocrystals were polymer-stabilized by irradiation at 365 nm with an intensity of 4 mW cm^− 2^.

### BP phase analysis

To characterize the phase behavior of the BP precursor mixtures, thermal cycling was performed using an Instec HCS402 hot stage equipped with an STC 20U controller. Samples were cooled from the isotropic phase at 1 °C/min. The emerging mesophases were monitored via polarized optical microscopy (POM) in reflection mode using an Olympus BX51 microscope equipped with a 5×/0.15 NA objective. For crystallographic assessment, Kossel patterns were recorded in conoscopic configuration using a 60×/0.70 NA objective under monochromatic illumination (450, and 550 nm). Reflection spectra were acquired using an Ocean Optics Flame-T spectrometer to evaluate photonic stopbands and correlate them with lattice parameters.

### Kossel analysis

Kossel pattern simulations were carried out using a diffraction modeling software (JCrystalSoft) that has been adapted for BPI and BPII structures. The program was modified to simulate Kossel-Kikuchi patterns of body-centered cubic (BCC) and simple cubic (SC) lattices corresponding to the BPI and BPII phases, respectively. Simulations accounted for converging monochromatic light sources at 550 nm and were used to generate orthographic projections of Kossel lines for various crystallographic orientations. By matching these simulated patterns to experimental Kossel images, we extracted accurate lattice constants, assuming an average refractive index of *n* = 1.6 for the BP material.

### Phase modulation of the BP crystal

The phase responses of the samples were tested using a Mach-Zehnder interferometer. The experimental setup consisted of the following devices: He-Ne Laser OptoSigma OSK-6328-18P, Waveform generator Agilent 33,220 A, Amplifier FLC Electronics A400D, and CMOS camera BASLER acA2500-60 μm. The wrapped phase extraction followed the five-frame temporal phase shift method^15^. Phase retrieval was performed using a fast two-dimensional phase-unwrapping algorithm^16^. Acquiring the value of the phase shift for each voltage required using reference areas in the interferograms, allowing for an increased system stability^17^. The data analysis enabled the determination of voltage-dependent phase-shift values at each point across the sample area. For this study, we report the mean phase-shift values averaged over the entire active area of the sample. The uncertainty of this mean was evaluated using the type B measurement uncertainty formula:

$$\:u\left(\bar{\varphi\:}\right)=\sqrt{{{\sigma\:}_{\bar{\varphi\:}}}^{2}+\frac{{\left({\Delta\:}\varphi\:\right)}^{2}}{n}}$$

where: Δφ is the error of the single phase measurement estimated as λ/20^18^ and $\:{\sigma\:}_{\bar{\varphi\:}}$ is the standard deviation of the mean phase value, which can be represented as:

$$\:{\sigma\:}_{\bar{\varphi\:}}=\sqrt{\frac{\sum\:_{i=1}^{n}{({\varphi\:}_{i}-\bar{\varphi\:})}^{2}}{(n-1)n}}$$

where *n* is the number of calculated points.

### Reflection spectra

Reflection spectra were acquired using a broadband white-light illumination setup. A broadband lamp integrated into the microscope served as the light source, and the reflected signal was collected via a fiber-coupled Ocean Optics Flame-T spectrometer. The incident beam was focused normally onto the BP cell with a spot size of approximately 1 mm, and the reflected light was collected through the same optical path. Prior to each measurement session, the spectrometer was wavelength-calibrated using the reference lamp.

The BP samples were mounted in a temperature-controlled stage to maintain lattice stability during irradiation. For photoisomerization experiments, a 365 nm LED was used as the excitation source, operated independently of the probe beam to allow subtraction of its contribution. No time-resolved pump–probe detection was employed; instead, steady-state reflection spectra were recorded as a function of irradiation time and intensity to monitor reversible photonic bandgap shifts.

## Supplementary Information

Below is the link to the electronic supplementary material.

Supplementary Material 1

## Data Availability

All relevant data and materials are addressed in this manuscript; data sets from optical measurements are available from the authors. Contact: Eva Oton – eva.oton@wat.edu.pl.
