# Supplementary material for: Optically isotropic fast phase modulation in 3D blue phase photonic crystals
Source: Sci Rep. 2025 Nov 25;15:41878. doi: 10.1038/s41598-025-25918-0 (PMC12647619; doi:10.1038/s41598-025-25918-0)
Supplement: Supplementary file 1 — Supplementary Material 1 [file 41598_2025_25918_MOESM1_ESM.docx]

**SUPPLEMENTARY INFORMATION**

**Optically Isotropic Fast Phase Modulation in 3D Blue Phase Photonic Crystals**

Tomasz Jankowski^1^, Eva Oton^1^, Noureddine Bennis^1^, Anna Pakuła^2^, Przemysław Morawiak^1^, Wiktor Piecek^1^,

^1^Faculty of New Technologies and Chemistry, Military University of Technology, Warsaw, Poland

^2^Faculty of Mechatronics, Warsaw University of Technology, Warsaw, Poland

Corresponding author: eva.oton@wat.edu.pl

List of Supplementary Materials:

1. Additional manufacturing information
2. Additional optical characterisation

1. **BP Monocrystals - additional manufacturing information**

BP precursor mixtures were prepared following our previously developed monocrystal fabrication protocol [9]. Large BP monocrystals with controlled lattice orientation and reflection wavelength were obtained by fine-tuning the chiral dopant (CD) concentration in the precursor and applying weak anchoring conditions using nylon alignment layers. The composition of a representative BP precursor mixture is provided in Fig. S1.

Uniform monodomain crystals were consistently achieved across macroscopic scales. Notably, lattice orientation was found to depend strongly on CD concentration even under identical surface treatments, suggesting that subtle variations in LC–substrate anchoring energy dictate orientation selection. Weak anchoring proved essential for this control, as conventional strong anchoring layers did not yield monodomain alignment. Furthermore, systematic analysis of BPII-to-BPI phase transitions revealed reproducible, symmetry-constrained orientation pathways and the absence of certain transitions, pointing to underlying crystallographic restrictions between the simple cubic (BPII) and body-centered cubic (BPI) phases. Based on these insights, we constructed a practical mapping between CD concentration, helical twisting power, and resulting BP orientation. This mapping not only facilitates rational dopant design for targeted orientations but also enables orientation identification without relying on complex Kossel pattern analysis [10].


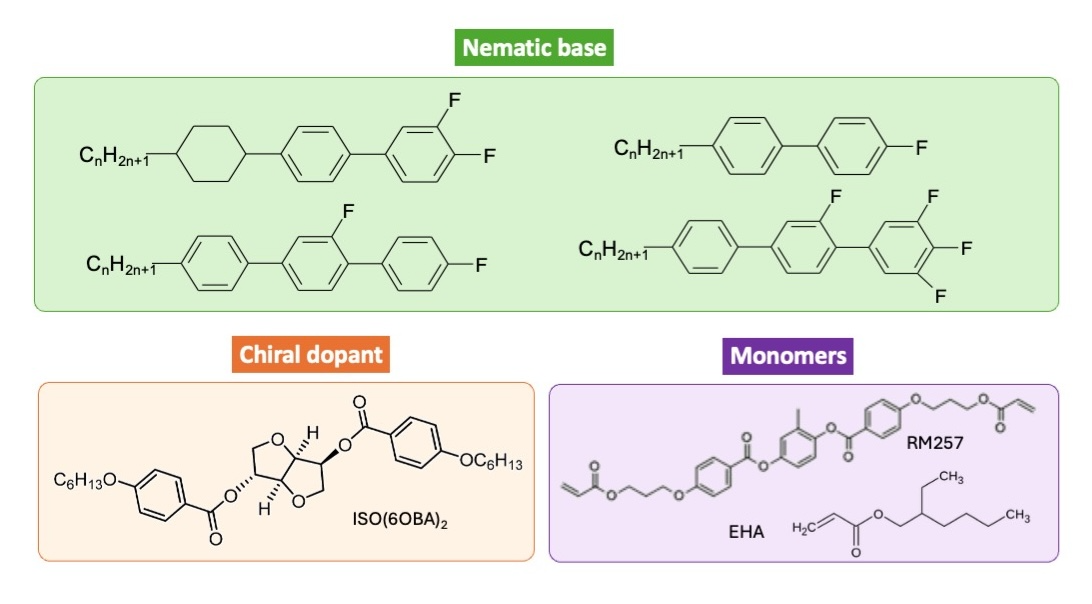


*Figure S1. Precursor mixture components*

1. **BP samples - additional optical characterisation**

To evaluate intensity changes accompanying phase modulation, the samples were illuminated with a spatially filtered, collimated 632.8 nm He-Ne laser beam. The beam passed through a quarter-wave plate and a rotatable polarizer, allowing three distinct states of linear polarization to be studied. Transmission was recorded with a CMOS camera while systematically varying the applied voltage. Mean pixel values across the active region were normalized to reference images obtained without the sample.

| 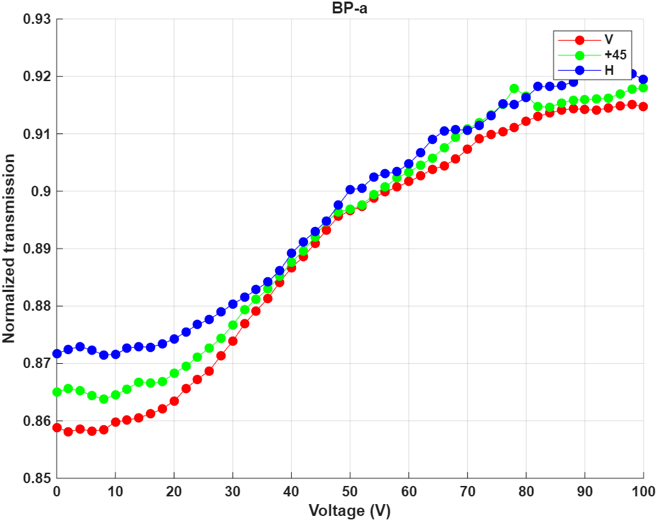 | 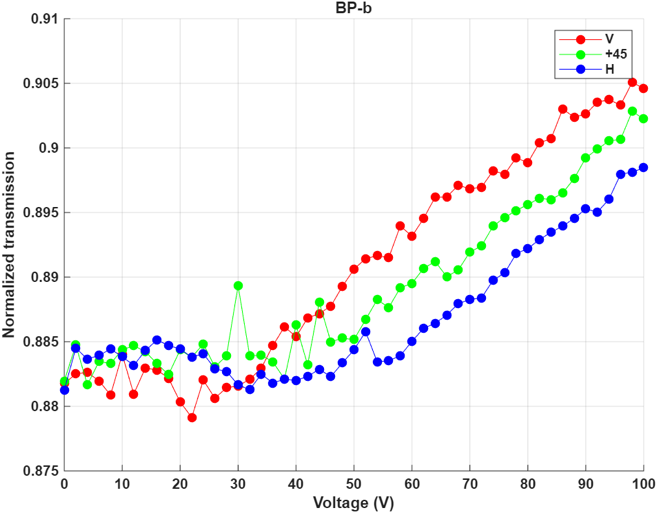 |
| --- | --- |

*Figure S2. Intensity modulation for BP-a and BP-b with different polarization states of the incident beam*

Both BP samples showed a modest increase in transmission with voltage, consistent with reduced scattering effects. The observed variation in transmitted intensity was ~6% for BP-a and ~2.5% for BP-b.

To quantify the trade-off between phase modulation and transmission changes, we defined a figure of merit (FoM) as the ratio of maximum phase shift (Δφ) to the corresponding normalized intensity change (ΔI). For a phase shift of 1 rad, the FoM was ~17 rad per unit ΔI in BP-a and ~40 rad per unit ΔI in BP-b.
